# London Ambulance: "Benefits Forgot"

**Published:** 1894-03-24

**Authors:** 


### The Hospital

An Institutional Journal of

The Medical Sciences and Hospital Administration, with

Vol. XV.—No. 391.] AN EXTRA NURSING SUPPLEMENT. [March 24, 1894.

### LONDON AMBULANCE: "BENEFITS FORGOT."

A special commissioner of the *Pall Mall Gazette* has contributed to that newspaper an account of the ambulance and first aid to the injured system which is carried out in Vienna under the controlling direction, and partly at the cost of Baron Mundy. That system appears to be very excellent and complete, and well worthy of being studied as an object lesson by other cities and towns less completely furnished with the resources of first aid. Baron Mundy is justly proud of his work, and like other practical enthusiasts, is very desirous of bringing the whole suffering world within the range of aid as benevolent, as prompt and as effective as that which is available at Vienna. This is exactly as it should be, and we sympathise as heartily with the Baron's propagandist enthusiasms as we congratulate him sincerely on the conspicuous success of his benevolent achievements.

But justice must be done to London as well as to Vienna; and if we have our own Baron Mundy, albeit a retiring man, whose name is only known to a few, he also should receive a due meed of recognition when occasion serves. Baron Mundy informed the *Pall Mall's* commissioner that "London is urgently in need of a first aid society, and why one has not been founded ere this is to me incomprehensible." His feeling, when discussing the subject, was that he should "at once proceed to London and do what was necessary himself"; and he was only prevented from carrying out this amiable project by the fact of his age and his invalid condition. In all this one recognises the impulses of a noble and generous mind; and though the Baron is misinformed on the question of London's ambulance necessities and the way they are provided for, the spontaneous enthusiasm which is ready to hasten to any part of the world in a great cause may well excite our admiration and provoke our imitation.

London is not "urgently in need of a first aid society." She has one—nay, she has several; and every one of them is well manned, well instructed, well equipped, and thoroughly efficient. The St. John Ambulance Association is familiar to every one, and its work needs no commendation. Similarly the hospitals under the control of the Local Government Board have their very complete horse ambulances, which are in full request every day and all the year round carrying the fevered sick to the infectious hospitals with all imaginable comfort and safety. The Royal Humane Society, which is really a department, and a very important department of first aid, has its hundreds of trained agents and its resources for resuscitation and recovery in every part of the metropolis and the kingdom. Of these we need not further speak, except to say that in Vienna all similar departments of ambulance and first aid seem to be combined under one system, and that system is placed under the enthusiastic and competent control of Baron Mundy. But in addition to all these there is in London an ambulance society which in the completest possible way endeavours to provide for every variety of street accident, and to secure the removal of every injured person to hospital or home without the delay of a single unnecessary moment. That organisation, whose initiator and actual founder desires to remain anonymous, is the work of the ambulance department of the Hospitals Association. It will surprise many persons—indeed, it will apparently surprise the whole British public, if the *Pall Mall's* commissioner is to be taken as the measure of the public's knowledge—to be informed that the ambulance department of the Hospitals Association has already provided no fewer than sixty-one fully-equipped hand ambulances for London alone. These sixty-one ambulances are distributed over every part of the metropolis within, and extending to the limits of the four-mile radius. In 1892, the report states that as many as 1,267 injured persons were transported to various hospitals by their means. When it is remembered that in that year only a little over three thousand persons required removal by street ambulance, it will be seen how large a proportion of the first aid and ambulance work of the metropolis is carried out by this excellent department. There still remains to be told the most gratifying fact of all in connection with this admirable work. That fact is that the whole outlay of these sixty-one ambulances has been borne by one philanthropic person. Mr. H. L. Bischoffscheim is the English Baron Mundy who has supplied the city of his adoption with this truly noble ambulance service at his own sole cost.

There are two or three points in connection with this little story of benevolence which well conditioned persons will be thankful to be reminded of. The first is that whilst Londoners have been profiting by the work of the Hospitals Association and the benevolence of Mr. H. L. Bischoffscheim to the number of nearly 1,300 persons annually for the last five years, they seem to have partly forgotten the existence of the Hospitals Association and entirely that of Mr. H. L. Bischoffscheim. That is not as it should be. Nevertheless it seems to be certainly the fact; for if so unusually well-informed a person as the special commissioner of a great newspaper is entirely ignorant on the subject, how much more ignorant must be the average man in the street? Shakespeare, our greatest Englishman, held very strong opinions on the subject of ingratitude. Nothing, he considered, showed such baseness of character as "ingratitude" and "benefits forgot." "Blow, blow, thou winter wind: thou art not so unkind as man's ingratitude." "How sharper than a serpent's tooth it is to have a thankless child." This is a cynical age we are all conscious, but if it becomes an ungrateful age it will be worse than cynical; it will be detestable, and by future ages its records will be detested. One would rather show gratitude to ten undeserving men than fail of it to a single one who is really worthy of it. Mr. H. L. Bischoffscheim has deserved the profound gratitude of all London. He will not suffer, but London will if that gratitude is not duly paid, by at least an honourable recognition. That is our story's first practical moral.

There is another moral, and, from the point of view of the self-interest of Londoners, it is still more important. Ambulances will wear out; our city and its suburbs will grow larger. What was enough for last year will not be sufficient for next. Mr. Bischoffscheim will not live for ever; and even if he should he might not be able or willing to put his hand deep into his pocket every two or three years to replace old ambulances or to supply additional ones for our extending streets and growing population. He has laid the foundation: it is for us to build upon it. He has created a corps: it is for us to develop it into an ever-increasingly efficient standing army. The London of to-day must have ambulances; the London of next century will in all probability need them very much more. Here is an organisation in existence with what business people call a "working plant." It will be an easy work to carry it on and increase it on its present lines; it would be a very difficult work to create it afresh if it were allowed to die out, and to rebuild it again from its foundations. The Ambulance Society of the Hospitals Association has its own Honorary Secretary (Mr. Thomas Ryan, of St. Mary's Hospital), its own office, and its own organisation, as well as its costly "plant" in full and efficient order. What is to be its future? A year or two ago the London County Council contemplated the taking of it over, and giving it that full and complete development which municipal activity and municipal resources can so easily promote. If that idea is not to be carried out, the thoughtful public should make itself fully acquainted with the history and working of the association, and should give it a place among those approved, permanent, and indispensable organizations which are worthy of constant and adequate support. Our object in writing this article has been twofold: first, to remind Londoners of the gratitude they owe, but have forgotten to pay, to one of their most self-hiding philanthropists; and, secondly, to make them acquainted with the noble ambulance service which has been started for them, and to ask them, through the County Council, to provide promptly and generously for its efficient maintenance and growth.

